# Peripheral Pathways to Neurovascular Unit Dysfunction, Cognitive Impairment, and Alzheimer’s Disease

**DOI:** 10.3389/fnagi.2022.858429

**Published:** 2022-04-18

**Authors:** Amy R. Nelson

**Affiliations:** Department of Physiology and Cell Biology, College of Medicine, University of South Alabama, Mobile, AL, United States

**Keywords:** Alzheimer’s disease, neurovascular dysfunction, infection, modifiable risk factors, germs, peripheral amyloid hypothesis

## Abstract

Alzheimer’s disease (AD) is the most common form of dementia. It was first described more than a century ago, and scientists are acquiring new data and learning novel information about the disease every day. Although there are nuances and details continuously being unraveled, many key players were identified in the early 1900’s by Dr. Oskar Fischer and Dr. Alois Alzheimer, including amyloid-beta (Aβ), tau, vascular abnormalities, gliosis, and a possible role of infections. More recently, there has been growing interest in and appreciation for neurovascular unit dysfunction that occurs early in mild cognitive impairment (MCI) before and independent of Aβ and tau brain accumulation. In the last decade, evidence that Aβ and tau oligomers are antimicrobial peptides generated in response to infection has expanded our knowledge and challenged preconceived notions. The concept that pathogenic germs cause infections generating an innate immune response (e.g., Aβ and tau produced by peripheral organs) that is associated with incident dementia is worthwhile considering in the context of sporadic AD with an unknown root cause. Therefore, the peripheral amyloid hypothesis to cognitive impairment and AD is proposed and remains to be vetted by future research. Meanwhile, humans remain complex variable organisms with individual risk factors that define their immune status, neurovascular function, and neuronal plasticity. In this focused review, the idea that infections and organ dysfunction contribute to Alzheimer’s disease, through the generation of peripheral amyloids and/or neurovascular unit dysfunction will be explored and discussed. Ultimately, many questions remain to be answered and critical areas of future exploration are highlighted.

## Introduction

What is Alzheimer’s disease (AD)? The answer to this question is challenging and varies significantly depending on who you ask; whether it’s a medical or scientific expert or a caregiver to an AD patient. Is AD only about cerebrospinal fluid (CSF) biomarkers of amyloid-beta (Aβ), tau, and neurodegeneration? These biomarkers have been suggested to be key diagnostic criteria to defining AD although, at the same time, Abeta plaques and neurofibrillary deposits were acknowledged as perhaps not causal to AD pathogenesis (Jack et al., 2018). Upon asking a caregiver this question, the response will likely involve memory impairment and the associated complications of daily living. Is it possible to define AD without knowing the cause? For this review, the definition of AD includes clinical symptoms such as cognitive impairment, including loss of recent memory and language ability, impairment of orientation, problem solving, and abstract thinking.

What causes AD and how do we stop it? Scientists learn how to formulate and rigorously test a hypothesis, how to design experiments to test this hypothesis and how to discern correlation from causation. If there is a fallen tree in the forest that has a mushroom on it, did the fungus cause the tree to die? Likewise, if there are Aβ plaques and tau tangles in the brain of a person with no cognitive deficits, do they cause AD? Often, but not always, Aβ plaques, neurofibrillary tau tangles and other amyloid neuropathologies are abundant in the brain of AD patients. Why and where were these amyloids produced and what leads to their accumulation in the brain? Here, the literature related to neuropathological, cellular, and subcellular changes and their role in AD are evaluated.

When thinking about root causes of AD, it might be helpful to consider the overarching umbrella of dementia as an entity. From this perspective, there are three forms of dementia: (1) sporadic neurodegenerative diseases (e.g., AD, Parkinson’s disease, multiple sclerosis, frontotemporal lobar dementia, amyotrophic lateral sclerosis (ALS), etc.), (2) genetic forms of dementia (e.g., autosomal-dominant AD (ADAD), Huntington’s disease, etc.), and (3) incident dementia [e.g., human immunodeficiency virus-1 (HIV-1)-associated neurocognitive disorders, long-term cognitive impairment after respiratory failure or shock]. It is unclear if “incidents” like infection or organ dysfunction could cause or contribute to sporadic dementias, like AD.

In the AD field, when reading “amyloid” it is often thought to mean “Aβ.” However, the definition of an amyloid is any polypeptide that polymerizes to form a cross-β structures. Classically, they have been stained in histopathology using dyes such as Congo red. There are many amyloids implicated in neurodegenerative diseases including not only Aβ, but also tau, transactive response DNA and RNA binding protein 43 kDa (TDP-43), α-synuclein, and superoxide dismutase-1. Due to their cross-β sheet structure, all amyloid monomers are capable of aggregating to form oligomers and fibrils (Verma et al., 2015). Amyloid oligomers are soluble, whereas fibrils are larger and insoluble. Neuropathological accumulates such as tangles, plaques, or Lewy bodies are formed by highly ordered amyloid fibrils. Evidence supports that oligomeric amyloids may represent the primary pathogenic structure (Glabe, 2006). Many types of amyloids co-exist in AD. Recent studies found that cerebral amyloid angiopathy (CAA), limbic-predominant age-related TDP-43 encephalopathy (LATE), and Lewy bodies all accumulate alongside Aβ plaques and tau tangles (Robinson et al., 2021). Many amyloids have prion-like seeding and spread (Holmes and Diamond, 2014). The role of amyloids in innate immune responses and as antimicrobial peptides will be detailed below in section “The Battle Between Humans and Germs.”

Is there only one cause of AD or is it a multifactorial syndrome? Humans and their unique genetic makeup, sex, ethnicity, environment, lifestyle factors, etc. make determining the cause of many human ailments challenging. Beyond humans themselves, there is mounting evidence that the 39 trillion germs residing in the human body play either beneficial and/or pathogenic roles that impact cognitive function. The strongest evidence currently impacting the globe and demonstrating that microorganisms can lead to cognitive impairment is the brain fog associated with severe acute respiratory syndrome coronavirus 2 (SARS-CoV-2). Recent developments regarding how the interplay between human and microbes contributes to incident dementia and perhaps AD have been quite intriguing. An individual’s impact (self) and the role germs (non-self) play and the possible relationships between these in the development of AD will be discussed.

## An Accelerated Historical Perspective of Alzheimer’s Disease Neuropathology

**FIGURE 1:**
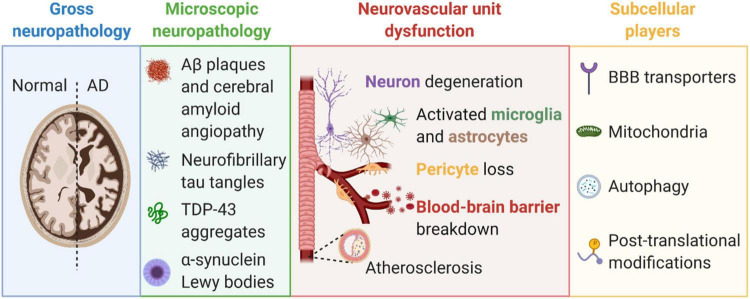
Alzheimer’s disease is characterized by gross pathologies (e.g., brain atrophy and ventricular enlargement, blue box), microscopic neuropathologies (e.g., amyloid-β plaques, tau tangles, and TDP-43 aggregates, green box), neurovascular unit dysfunction (e.g., blood-brain barrier breakdown, pericyte loss, neurodegeneration, and gliosis, pink box), and changes to subcellular players (e.g., blood-brain barrier transporters, mitochondria, autophagy, and post-translational modifications, yellow box). Created with BioRender.com.

Scientific discoveries described 115 years ago by Dr. Oskar Fischer (Eikelenboom et al., 2006; Goedert, 2009) and Dr. Alois Alzheimer (Alzheimer et al., 1995) provided the fundamental basis of what we know about AD neuropathology today. Notably, Dr. Fischer described neuritic plaques in 12 cases of dementia (Goedert, 2009) and described that the crucial step in plaque formation involves deposition of a foreign substance, provoking an inflammatory reaction, and followed by degeneration around nerve fibers (Eikelenboom et al., 2006). Dr. Alzheimer described histological alterations in the brain of dementia patient August D. as including vascular arteriosclerotic changes, neurofibrils positive for Bielschowsky’s silver method that appeared where neurons used to be, development of fibers and adipose saccules in glia, growths on endothelial cells, and proliferation of vessels (Alzheimer et al., 1995). Over the last century, many great scientists have continued to expand our knowledge about neuropathological changes in the AD brain. Today, it is broadly accepted that AD brains encompass gross neuropathology including brain atrophy and enlargement of ventricles, microscopic amyloid neuropathologies (e.g., Aβ plaques, CAA, neurofibrillary tau tangles, Lewy body α-synuclein pathology, and TDP-43 aggregates), neurovascular unit dysfunction [e.g., pericyte loss, blood-brain barrier (BBB) breakdown], and altered subcellular players (e.g., BBB transporter expression changes, post-translational modifications) (Figure 1). Amyloids implicated in AD are described below in more detail.

### Aβ

Aβ are peptides of various lengths (36–43 amino acids) are formed by the cleavage of amyloid precursor protein (APP) by β- and γ-secretases. Aβ_1–40_ and Aβ_1–42_ are the most studied species. *APP* is expressed in various cell types in all tissues and organs as reported in the Human Protein Atlas.^1^ Aβ peptides can aggregate generating various forms of soluble oligomers. Misfolded Aβ oligomers have prion-like properties as they can induce other Aβ molecules to misfold. Experimental studies have shown that prion protein antagonists rescue Aβ-related synaptic and cognitive deficits (Gunther et al., 2019; Sagare et al., 2019). Aβ is also an antimicrobial peptide as described below in section “Antimicrobial Peptides.”

The “amyloid cascade hypothesis” has been the central focus of AD research for nearly 30 years (Selkoe and Hardy, 2016). This hypothesis initiated with the presence of Aβ plaques in the brain of many AD patients, and gained strength and momentum as carriers with genetic mutations in *APP* and presenilin (*PSEN*) were found to have increased Aβ production and develop autosomal dominant AD, and individuals with Down’s syndrome have three copies of *APP*, develop Aβ plaques and many develop dementia (Kolata, 1985; Selkoe and Hardy, 2016). Furthermore, apolipoprotein E4 (*APOE* ε*4*) increases the risk of sporadic AD and reduces clearance of Aβ from brain (Shibata et al., 2000; Deane et al., 2008; Ramanathan et al., 2015). Aβ oligomers decrease synapse density and reduce long-term potentiation, a cellular correlate to learning and memory, in rodent brain slices in some studies (Selkoe and Hardy, 2016).

Evidence disproving the amyloid cascade hypothesis has been increasing. Not all people with plaques and increased Aβ expression develop AD, including those with Down’s Syndrome (Zigman et al., 2008). In a recent study of 747 individuals, subtle cognitive difficulties associated with entorhinal cortex atrophy were present prior to amyloid plaque formation (Thomas et al., 2020). Recent estimates show that more than a third of cognitively normal people over the age of 70 and more than a half of individuals over the age of 95 have elevated Aβ_42_ in their CSF (Jansen et al., 2022). Most disappointingly, more than 30 Phase three clinical trials of drugs targeting Aβ have failed to slow cognitive decline in AD (Ayton and Bush, 2020).

Some studies have suggested that the link between Aβ plaques and cognitive deficits might be due to immune status. For example, increased plasma levels of the proinflammatory cytokines interleukin-12 and interferon-γ predict improved cognition among elderly subjects with Aβ detected by neuroimaging (Yang et al., 2021), suggesting that peripheral immune status may be the key between Aβ plaque presence and cognitive impairment. Furthermore, central immune status may also be critical as microglia-associated genes such as triggering receptor expressed on myeloid cells 2 (TREM2) and CD33 have been implicated in AD, as detailed below in sections “Triggering Receptor Expressed on Myeloid Cells 2 and Others.”

Interestingly, *APP* and *PSEN* mutations cause neurovascular abnormalities as described below in the section “Genetics.”

### Tau

*MAPT* encodes for tau. *MAPT* is expressed in various cell types in most tissues and organs (e.g., brain, heart, kidney, lung, skeletal muscle, etc.) as reported in the Human Protein Atlas (see footnote 1). Tau aggregates and inclusions occur in multiple neurodegenerative diseases including not only AD, but also progressive supranuclear palsy, corticobasal degeneration, Pick’s disease, chronic traumatic encephalopathy, frontotemporal dementia with parkinsonism, and many others (Strang et al., 2019). Neurofibrillary tangles are also present during normal aging and has been defined as “primary age-related tauopathy” (PART) (Crary et al., 2014). Diseases in which tau aggregates are referred to as tauopathies (Strang et al., 2019). More than 50 pathogenic mutations in *MAPT* have been reported and linked to tauopathies (Strang et al., 2019).

In the brain, tau provides stability to microtubules for axonal transport (Ballatore et al., 2007). There are many exon splice variants and isoforms or “strains” of tau in the brain and periphery all being dynamically regulated by post-translational modifications (e.g., phosphorylations, glycosylations, ubiquitinations, glycations, nitrations, oxidations, etc.) (Strang et al., 2019). In addition, tissue transglutaminase cross-links tau into insoluble filamentous structures (Appelt and Balin, 1997; Balin and Appelt, 2000). Recent studies have suggested that misfolded four-repeat (4R) tau is associated with rapid progression of AD (Kim et al., 2022).

In AD, tau no longer binds to microtubules and becomes sequestered into neurofibrillary tangles in neurons and glia. Neurofibrillary tau tangles have a routine pattern of spread that has allowed for assessing the stage of AD, also known as Braak stages (Hyman et al., 1984; Braak and Braak, 1991; de Calignon et al., 2012). There is evidence that this spread occurs through a combinatorial process of synaptic or exosomal secretion followed by endocytic or exosomal uptake (Strang et al., 2019). Some experimental studies, but not all, have found tau aggregation and spreading in cell culture and animal models, primarily those with humanized tau (e.g., hTauP301S) (Strang et al., 2019). Tau tangles are comprised of multiple types of tau isoforms (Goedert et al., 1989), and are thought to have both a loss-of-function (e.g., microtubule stabilization) and toxic gain-of-function in AD (Ballatore et al., 2007). Previous animal studies found that reducing endogenous tau levels prevented behavioral deficits and excitotoxicity in transgenic mice that overexpress Aβ without changing Aβ levels (Roberson et al., 2007). Tau lesions have been demonstrated to accumulate prior to Aβ in AD and better correlate with cognitive impairment (Johnson et al., 2016). However, in a three-dimensional human AD neural cell culture model, Aβ induced the production of phosphorylated tau aggregates (Choi et al., 2014).

Strikingly, several studies have shown that toxic tau is transmissible (Clavaguera et al., 2009, 2013; Nonaka et al., 2010), leading to the idea that progression of the diverse but characteristic tau pathologies occurs through prion-like seed-dependent aggregation (Hasegawa, 2016; Goedert and Spillantini, 2017). Importantly, transmissible tau can be generated not only in the brain but also by peripheral organs in response to an infection, including lung endothelial cells (Balczon et al., 2013, 2017, 2019; Choi et al., 2021), and has been shown to reduce dendritic spine density (Scott et al., 2020), impair learning and memory (Balczon et al., 2019), and cause neuronal tauopathy (Choi et al., 2021). The role of peripheral infections or inflammation leading to brain tau tangles is an understudied and vital area of research.

### α-Synuclein

*SNCA* encodes for α-synuclein. *SNCA* is expressed in a few cell types in many tissues and organs (e.g., brain, heart, kidney, lung, skeletal muscle, etc.) as reported in the Human Protein Atlas (see footnote 1). Mutations in *SNCA* have been implicated with parkinsonism. α-synuclein aggregates to form insoluble fibrils in Lewy bodies. Lewy bodies are neuropathologies commonly associated with Parkinson’s disease and dementia with Lewy bodies. Lewy bodies are also present in AD brain in proximity to Aβ plaques and tau tangles (Robinson et al., 2021). These disorders are known as synucleinopathies. There are at least three isoforms of α-synuclein produced through alternative splicing. The most investigated is the full-length 140 amino acid protein. α-synuclein, like the aforementioned amyloids, can propagate in a prion-like manner (Holmes and Diamond, 2014). Upregulation of α-synuclein in response to an immune activation was recently reviewed in the context of Parkinson’s disease (Kasen et al., 2022; Linard et al., 2022).

### Transactive Response DNA and RNA Binding Protein 43 kDa

*TARDBP* encodes for TDP-43. *TARDBP* is expressed in many cell types in all tissues and organs as reported in the Human Protein Atlas (see footnote 1). TDP-43 is involved in the regulation of RNA splicing, stability, transcriptional repression, and other cellular functions (Huang et al., 2020). TDP-43 proteinopathy was first identified in ALS and frontotemporal lobar degeneration (Neumann et al., 2006). However, TDP-43 neuropathology was also identified in post-mortem AD brain (Amador-Ortiz et al., 2007; Uryu et al., 2008; Josephs et al., 2014, 2016), and is often observed in older adults with LATE (Nelson et al., 2019; Katsumata et al., 2020; Robinson et al., 2020; Uemura et al., 2022). TDP-43 frequently coexists with tau and α-synuclein in brain tissue of subjects with AD and dementia with Lewy bodies (Higashi et al., 2007).

TDP-43 neuropathology had strong associations with cognition, memory loss, and medial temporal atrophy in AD (Josephs et al., 2014). Similar to tau, TDP-43 has staging and spread with deposition beginning in the amygdala, then moving to entorhinal cortex and subiculum; to the dentate gyrus of the hippocampus and occipitotemporal cortex; insular cortex, ventral striatum, basal forebrain, and inferior temporal cortex; substantia nigra, inferior olive and midbrain tectum; and finally to basal ganglia and middle frontal cortex (Josephs et al., 2016). TDP-43 has been demonstrated to have prion-like seeding and propagation (Furukawa et al., 2011). Mitochondria-associated TDP-43 is increased in AD patients and transgenic mice for AD (Gao et al., 2020). Suppression of TDP-43 prevents neuronal loss and improves cognitive and motor function in 5XFAD transgenic mice (Gao et al., 2020).

Lipopolysaccharide-induced inflammation promotes TDP-43 mislocalization and aggregation suggesting that inflammation may contribute to the development and exacerbation of TDP-43 in AD and other neurodegenerative diseases (Correia et al., 2015). How peripheral insults lead to TDP-43 pathologies in AD remains to be determined.

## Neurovascular Unit Dysfunction in Alzheimer’s Disease

The neurovascular unit is comprised of endothelial cells, mural cells (e.g., pericytes and smooth muscle cells), glia (e.g., astrocytes and microglia), and neurons (Figure 1) that precisely regulate cerebral blood flow (CBF) to assure that brain energy demands are met (Zlokovic, 2011; Zhao et al., 2015a; Nelson et al., 2016b; Kisler et al., 2017a; Sweeney et al., 2019b). There is growing appreciation and strong evidence that neurovascular uncoupling, CBF reductions and dysregulation, and breakdown of the BBB, including the loss of pericytes, are early events in the AD pathophysiological cascade (Iadecola, 2004, 2013; Zlokovic, 2011; Toledo et al., 2013; Montagne et al., 2015, 2020; Sweeney et al., 2015, 2018a,b, 2019a,b; Zhao et al., 2015a; Arvanitakis et al., 2016; Halliday et al., 2016; Iturria-Medina et al., 2016; Nelson et al., 2016b; Kisler et al., 2017a; Kirabali et al., 2019; Miners et al., 2019; Nation et al., 2019; Bourassa et al., 2020; Shi et al., 2020), and are independent of Aβ and tau alterations in the brain (Nation et al., 2019; Montagne et al., 2020). However, Aβ oligomers induce vasoconstriction reducing CBF (Thomas et al., 1996; Nortley et al., 2019), and thus likely play a critical role in neurovascular unit dysfunction in AD. Improperly functioning blood vessels will fail to deliver essential nutrients, including oxygen and glucose to, and remove metabolic waste products from the brain (Zlokovic, 2011; Nelson et al., 2016b). If CBF is halted, brain functions will cease in seconds and permanent brain damage occurs in minutes (Moskowitz et al., 2010). Below, cells of the neurovascular unit are described and how they are altered in AD and with infections is discussed.

### Endothelial Cells and the Blood-Brain Barrier

The BBB inhibits uncontrolled entry of blood-derived products and pathogens from entering the brain and regulates molecule entry into and out of the brain by a specialized substrate-specific transport system (Zlokovic, 2011; Zhao et al., 2015a; Nelson et al., 2016b). Several studies used dynamic contrast enhanced magnetic resonance imaging (DCE-MRI) to quantify BBB permeability (BBB_*ktrans*_) and found increased BBB breakdown in individuals with normal aging (Montagne et al., 2015; Verheggen et al., 2020a,b), MCI (Montagne et al., 2015, 2020; Nation et al., 2019; Rensma et al., 2020), and early-AD (van de Haar et al., 2016, 2017a,b). T2*- and/or susceptibility weighted imaging MRI sequences have identified perivascular hemosiderin deposits/microbleeds in MCI (Yates et al., 2014) and individuals with early AD (Goos et al., 2009; Brundel et al., 2012; Uetani et al., 2013; Heringa et al., 2014; Olazarán et al., 2014; Yates et al., 2014; Zonneveld et al., 2014; Shams et al., 2015; Poliakova et al., 2016). Post-mortem human studies have shown reduced capillary length and microvascular degeneration with diminished tight junction proteins, basement membrane irregularities, and brain endothelial degeneration (Salloway et al., 2002; Bailey et al., 2004; Wu et al., 2005; Baloyannis and Baloyannis, 2012; Sengillo et al., 2013; Halliday et al., 2016). Additionally, capillary leakages of blood-derived products including fibrin(ogen), immunoglobulin G (IgG), thrombin, albumin and hemosiderin have been detected in the cortex, and hippocampus of AD post-mortem brain tissues (Cullen et al., 2005; Zipser et al., 2007; Ryu and McLarnon, 2009; Hultman et al., 2013; Sengillo et al., 2013; Cortes-Canteli et al., 2015; Halliday et al., 2016; Miners et al., 2018). Systemic infections and inflammation often increase BBB permeability allowing immune mediators and cells to enter the brain (Prinz and Priller, 2017).

### Brain Pericytes

Pericytes are critical for the maintenance of the BBB and have many other functions including angiogenesis, clearance of toxic metabolites (Ma et al., 2018), capillary hemodynamic responses (Kisler et al., 2017a,b; Nelson et al., 2020), neuroinflammation, and pluripotent stem cell activity (Sweeney et al., 2016). Pericyte injury marker soluble platelet derived growth factor β (sPDGFRβ) is elevated in cerebral spinal fluid in MCI and early AD (Montagne et al., 2015, 2020; Nation et al., 2019). Pericyte loss has been suggested using electron microscopy of AD cortex (Farkas and Luiten, 2001; Baloyannis and Baloyannis, 2012) and by decreased levels of pericyte marker PDGFRβ in the precuneus and underlying white matter (Miners et al., 2018). By immunostaining, pericyte number and coverage of brain capillaries were reduced in AD cortex and hippocampus compared to control brain (Sengillo et al., 2013), and this loss was accelerated in *APOE* ε*4* carriers (Halliday et al., 2016). Recent studies have shown that pericyte loss in the parietal cortex in AD brain correlates with cognitive decline and TDP-43 pathology (Bourassa et al., 2020).

Recent single nuclei RNAseq studies of vascular cells identified two novel types of pericytes (e.g., transporter- and matrix-type pericytes) in human post-mortem brain and identified a reduction of matrix-type pericytes in AD hippocampus and cortex (Yang et al., 2022). The consequence of the specific loss of matrix-type pericytes on neurovascular dysfunction in AD has yet to be determined.

Pericytes are the first line of brain defense when BBB integrity is compromised. They play a role in the removal of Aβ via the low-density lipoprotein receptor-related protein 1 (LRP1) (Ma et al., 2018). Pericytes are a target of infections such as HIV-1 (Bertrand et al., 2019), and SARS-CoV-2 (Bocci et al., 2021). SARS-CoV-2 enters pericytes via its spike protein and the angiotensin-converting enzyme-2 receptor (Khaddaj-Mallat et al., 2021; McCracken et al., 2021). Pericytes may protect the brain by clearing microorganisms and peripherally produced amyloids (e.g., Aβ, tau, and others) attempting to enter the brain. It remains to be determined how microorganisms and peripheral amyloids impact pericyte functions, especially their regulation of red blood cell flow and support of the BBB.

### Astrocytes and Microglia

Astrocytes regulate many dynamic processes in the brain including maintaining systemic homeostasis (e.g., pH, neurotransmitters, trophic factors, and calcium), modulating neuronal activity and plasticity, clearance of molecules from the brain, and their endfeet structurally support the BBB and neurovascular unit (De Strooper and Karran, 2016; Heithoff et al., 2021). Importantly, astrocytes mediate neurovascular signaling to pericytes contributing to CBF at the capillary level (Mishra et al., 2016). Astrocytes highly express APOE and secrete it to signal pericytes via LRP1 to suppress the activations of the cyclophilin A-matrix metalloproteinase-9 pathway that degrades the BBB (Bell et al., 2012). Preclinical (Bell et al., 2012; Jackson et al., 2021) and clinical studies (Montagne et al., 2020) have shown that APOE ε4 leads to BBB breakdown, as further detailed in section “Apolipoprotein E.”

Studies have demonstrated reactive astrocytes in post-mortem AD brain tissue using immunostaining (Beach and McGeer, 1988; Griffin et al., 1989; Meda et al., 2001), and around Aβ plaques in post-mortem brain tissue from aged subjects (Simpson et al., 2010). Using positron emission tomography to measure astrocytic marker monoamine oxidase B, studies found astrocytosis in the brain of MCI and early-AD participants (Carter et al., 2012). Activated astrocytes produce vascular endothelial growth factor-A that is associated with BBB breakdown in mice (Argaw et al., 2012).

Microglia are the macrophages of the brain. Although the total number of microglia in the AD brain remains unchanged, there are increased numbers of reactive microglia in post-mortem AD brain tissue (McGeer et al., 1987; Akiyama and McGeer, 1990; Cras et al., 1990; Styren et al., 1990). Microglia are able to bind Aβ oligomers and fibrils via cell surface receptors (Paresce et al., 1996; Bamberger et al., 2003; Liu et al., 2005; Stewart et al., 2010) and may be part of the inflammatory response observed in AD (Heneka et al., 2015). Reactive microglia are often found associated with dense Aβ plaques (Itagaki et al., 1989; Mattiace et al., 1990; Mackenzie et al., 1995; Sasaki et al., 1997), but have also been demonstrated to be surrounding approximately half of diffuse Aβ plaques in human brain (Itagaki et al., 1989). Studies using transgenic *APP* mice with microglial ablation demonstrate that microglia are not required for the formation or maintenance of Aβ plaques or associated neuritic dystrophy (Grathwohl et al., 2009). However, more recent studies in *APP*/*PS1* mice demonstrated that microglia play a role in dense-core plaque formation (Huang et al., 2021). Using transcriptional single-cell sorting, recent studies identified a novel microglia type associated with neurodegenerative diseases (Keren-Shaul et al., 2017; Olah et al., 2020). The TREM2-APOE pathway drives the transcriptional phenotype of dysfunctional microglia in AD and other neurodegenerative diseases (Krasemann et al., 2017). Genome-wide association studies (GWAS) have identified AD risk genes *CD33* and *TREM2* as being linked to immune responses and microglia (Griciuc and Tanzi, 2021), as detailed below in sections “Triggering Receptor Expressed on Myeloid Cells 2 and Others.”

Like in AD and other neurodegenerative diseases, astrocytes and microglia become reactive in response to inflammation or infection. The interaction and communication between these two cell types are starting to be unraveled with the ever-increasing amount of -omics and functional studies and data (Liddelow et al., 2020).

### Neurons

Neurons in the brain can be classified by their morphology and functions, including the release of specific neurotransmitters. The most abundant neurotransmitters are glutamate and gamma-Aminobutyric acid (GABA). However, neurons often implicated in AD and other neurodegenerative diseases often release other neurotransmitters, as further described below.

Post-mortem histology studies that found reductions in cholinergic markers in the brain and neurons in the nucleus basalis of Meynert laid the foundation for the “cholinergic hypothesis of AD” (Bowen et al., 1976; Davies and Maloney, 1976; Mesulam, 1976; Whitehouse et al., 1982; Mann et al., 1984) and were the motivation for acetylcholinesterase inhibitor drug treatments used in AD patients. The loss of cholinergic innervation in AD is often associated with neurofibrillary tau tangles and Aβ plaques (Geula and Mesulam, 1995; Braak and Del Tredici, 2013; Mesulam, 2013). Also, cholinergic neurons that degenerate in AD rely on retrograde transport of nerve growth factor (NGF) from hippocampus and cortex for proper function (Mufson et al., 2008; Schliebs and Arendt, 2011; Cattaneo and Calissano, 2012; Triaca and Calissano, 2016), and an imbalance in proNGF to mature NGF has repeatedly been implicated in AD (Cuello and Bruno, 2007; Cuello et al., 2007; Iulita and Cuello, 2014). While clinical studies found that cholinesterase inhibitor therapies provided significant symptomatic improvement in AD patients (Summers et al., 1986; Hampel et al., 2018, 2019), the cognitive benefit has not been generally perceived as profound and the treatments are often accompanied by negative side effects. The argument has been made that improved dosing and treatment regimens with cholinesterase inhibitors may prove to be more beneficial (Hampel et al., 2018). The production and presence of sufficient acetylcholine in brain is critical for proper function. *APOE* ε*4* aged mice have reduced evoked acetylcholine release in hippocampus as compared to *APOE* ε*3* mice (Dolejší et al., 2016). Also, *APOE* ε*4* allele copy number showed an inverse relationship with residual brain choline acetyltransferase activity and nicotinic receptor binding sites in the hippocampus and temporal cortex of AD subjects (Poirier et al., 1995). Interestingly, increased dietary choline improves cognitive function, prevents age-related memory decline and protects against AD neuropathological changes (Blusztajn et al., 2017; ScienceDaily, 2020). Humans fed a choline-deficient diet had lymphocyte DNA damage and caspase-3-dependent apoptosis of lymphocytes (da Costa et al., 2006). Choline oxidation metabolites improve cognitive prognosis in *APOE* ε*4* carriers (Hildre et al., 2020).

Noradrenergic neurons of the locus coeruleus are some of the first neurons to form fibrillar tau (Braak and Del Tredici, 2012) and degenerate in AD (Iversen et al., 1983; Mann et al., 1984; Förstl et al., 1994; Matthews et al., 2002; Zarow et al., 2003; Liu et al., 2013). High-resolution fast spin-echo T1-weighted imaging showed decreased locus coeruleus density in AD patients compared to controls (Takahashi et al., 2015). While there is agreement in the field with regards to noradrenergic degeneration in locus coeruleus, the levels of norepinephrine have been variably reported as decreased, unchanged or increased, as recently reviewed (Gannon et al., 2015). Elevated cerebral spinal fluid levels of norepinephrine have been detected and implicated in causing increased agitation and aggression in AD patients (Raskind and Peskind, 1994; Elrod et al., 1997). One potential source of elevated norepinephrine is from noradrenergic sympathetic sprouting (Nelson et al., 2014). Alterations to other monoaminergic neurons in AD, including to serotonergic, adrenergic, histaminergic, and melatonergic, have been recently reviewed (Šimić et al., 2017).

## Subcellular Changes in Alzheimer’s Disease

### Blood-Brain Barrier Transporters

Blood-brain barrier transporters are essential for moving essential molecules in and out of the brain that are otherwise unable to cross the BBB. For example, 70–85% of Aβ is cleared from the brain by transvascular clearance across the BBB (Deane et al., 2008; Ramanathan et al., 2015; Nelson et al., 2016b,^2017^). Here, key BBB transporters altered in AD are described. Additional details can be found in previous reviews (Ramanathan et al., 2015; Nelson et al., 2016b; Sweeney et al., 2019b) and a book chapter (Nelson et al., 2016a). Whether microorganisms and/or virulence factors use BBB transporters to enter the brain has yet to be determined.

#### Glucose Transporter-1

Glucose Transporter-1 (GLUT1) is the primary brain endothelial transporter of glucose into the brain. There is a significant reduction of GLUT1 in cognitively normal individuals with genetic risk for AD and in early AD (Simpson et al., 1994; Liu et al., 2008). There is also an impairment in brain glucose utilization in brain regions impacted by AD (Mosconi, 2005). In addition to AD, GLUT1 deficiency syndrome, also known as De Vivo disease, is a rare genetic metabolic disorder caused by *SLC2A1* gene, is inherited as an autosomal dominant trait, and is associated with mild to severe cognitive impairment (Pearson et al., 2013).

#### Low-Density Lipoprotein Receptor-Related Protein 1

Lipoprotein Receptor-Related protein 1 (LRP1) is a receptor for Aβ (Shibata et al., 2000; Deane et al., 2008; Ramanathan et al., 2015; Nelson et al., 2016b,a, 2017) and tau (Rauch et al., 2020). Aβ is cleared across the BBB as a free peptide and/or bound to APOE ε2 and APOE ε3, but not ε4 (Shibata et al., 2000; Deane et al., 2008) via receptor-mediated transcytosis that is regulated by phosphatidylinositol binding clathrin assembly protein (PICALM) (Zhao et al., 2015b). In normal aging and AD, there is a significant reduction in LRP1 expression in brain endothelial cells (Donahue et al., 2006) and vascular smooth muscle cells (Bell et al., 2009; Kanekiyo et al., 2012, p. 1). The decrease of LRP1 in microvessels negatively correlates with an increase of Aβ in the brain (Donahue et al., 2006). Astrocytic LRP1 is also involved in Aβ uptake in the brain (Liu et al., 2017). A recent study found that tau uptake and spread is regulated by LRP1 (Rauch et al., 2020). The genetic interplay between LRP1 and tau increases AD risk (Vázquez-Higuera et al., 2009). Interestingly, LRP1 has been identified as a receptor for Rift Valley fever virus (Bopp et al., 2021) and the major virulent factor of *Clostridioides difficile*, toxin B (Guo et al., 2021).

#### Receptor for Advanced Glycation Endproducts

Receptor for Advanced Glycation Endproducts (RAGE) is the major Aβ influx receptor at the luminal side of the BBB that transports Aβ from the blood into the brain (Deane et al., 2003). RAGE is normally expressed at low levels at the BBB, but its expression is increased in normal aging and in AD brain endothelium and is associated with increased cerebrovascular and brain accumulation of Aβ (Yan et al., 1996; Silverberg et al., 2010; Deane et al., 2012).

#### Clusterin (Also Known as Apolipoprotein J)

Clusterin (CLU) is involved in the clearance of misfolded proteins, regulation of apoptosis, inflammation, atherosclerosis, and cancer (Nuutinen et al., 2009). *CLU* has been identified as a genetic risk factor for sporadic AD by several GWAS (Harold et al., 2009; Lambert et al., 2009; Carrasquillo et al., 2010; Corneveaux et al., 2010). CLU interacts with Aβ and regulates its clearance from brain via LRP2 (also known as megalin or gp330) (Zlokovic et al., 1994, 1996; Bell et al., 2007; Nelson et al., 2017). Studies have reported elevated blood levels of CLU in AD (Abdi et al., 2022), however it is unclear whether this increase is beneficial or detrimental. A recent study found that plasma levels of CLU are increased with exercise and that injecting CLU intravenously in mouse models of acute brain inflammation or AD had reduced neuroinflammatory gene expression (De Miguel et al., 2021).

It remains to be determined whether GLUT1, LRP1, RAGE, or CLU play a role in incident dementia. Interestingly, CLU gene expression is elevated in the lungs of SARS-CoV-2 patients (Singh et al., 2021). RAGE levels were significantly increased in intensive care unit SARS-CoV-2 patients compared to healthy controls (Passos et al., 2022), and RAGE has been implicated in COVID-19 morbidity and mortality (Sellegounder et al., 2021). Mice treated with the RAGE antagonist FPS-ZM1 had improved survival and reduced inflammation upon SARS-CoV-2 infection (Jessop et al., 2022).

### Mitochondria

Mitochondria, the powerhouse of the cell, are altered in aging and AD (Navarro and Boveris, 2007; Eckert et al., 2011; Swerdlow et al., 2017). Studies using *in situ* hybridization to mitochondrial DNA (mtDNA), immunocytochemistry of cytochrome oxidase, and morphometry of electron micrographs found that neurons with increased oxidative damage in AD have increased mtDNA and cytochrome oxidase in the neuronal cytoplasm and in vacuoles associated with lipofuscin (Hirai et al., 2001). Morphometric analyses showed that mitochondrial numbers were significantly reduced in Alzheimer’s disease (Hirai et al., 2001). Another study investigating mitochondria in AD post-mortem brain tissue found altered mitochondrial cristae, accumulation of osmiophilic material, and decrease in mitochondrial size (Baloyannis, 2006). Also in AD, there is a reduction in α-ketoglutarate dehydrogenase complex and of pyruvate dehydrogenase complex enzymes (Sorbi et al., 1983; Gibson et al., 1988, 1998). Cyclooxygenase activity is lower in brain and platelet mitochondria in AD patients (Swerdlow, 2012). Targeting mitochondrial α-F1-adenosine triphosphate synthase increased intracellular calcium leading to sustained calcium/calmodulin-dependent protein kinase kinase β-dependent activation of the 5′ adenosine monophosphate-activated protein kinase/mammalian target of rapamycin (AMPK/mTOR) pathway, a canonical longevity mechanism (Goldberg et al., 2018).

Interplay between mitochondria and AD neuropathologies and cellular changes have been reported. Recent studies found that mitochondria-associated TDP-43 is increased in AD patients and transgenic mice for AD (Gao et al., 2020). Also, hyperphosphorylated or aggregated tau prevents axonal transport of mitochondria to meet high energy demands and regulate calcium buffering of neurons (Cheng and Bai, 2018). Pathological tau impairs mitochondrial dynamics by regulating mitochondrial fission/fusion proteins (Cheng and Bai, 2018). Interestingly, mild inhibition of complex I mitigates cognitive decline in AD transgenic mice via AMPK activation, reduction of Aβ, pTau, glycogen synthase kinase-3β, and restoration of axonal trafficking (Zhang et al., 2015). However, a natural mitochondrial complex I inhibitor, annonacin, caused tau pathology in cultured neurons (Escobar-Khondiker et al., 2007). Also, non-glycosylated full-length and C-terminal truncated APP accumulates in protein import channels of mitochondria of human AD susceptible brain regions and neurons and directly correlated with mitochondrial dysfunction (Devi et al., 2006). Fragmented microglial mitochondria triggered an astrocytic response and propagated neurodegeneration (Joshi et al., 2019). Of significance, reduced CBF may induce oxidative stress, largely due to reactive oxygen species, and over time initiates mitochondrial failure (Aliev et al., 2009; Parodi-Rullán et al., 2019).

### Autophagy

Autophagy is a lysosome-dependent, homeostatic mechanism of cells that degrades unnecessary or dysfunctional organelles and other proteins, and recycles them into energy. Autophagy or autophagy proteins interact with other cellular processes such as apoptosis, secretion, and endocytic pathways. PSEN1 is required for lysosomal acidification and protein degradation (Lee et al., 2010). Moreover, aberrant tau appears to disrupt axonal vesicle transport by impairing the dynein-dynactin complex, increasing the number of autophagosomes, and contributing to tau-induced toxicity in AD (Butzlaff et al., 2015). Aβ may be degraded by autophagy, and upregulation of autophagy has been shown to reduce Aβ levels (Boland et al., 2008; Spilman et al., 2010; Tian et al., 2011; Vingtdeux et al., 2011). Recent studies have implicated an interplay between autophagy and inflammasomes (Biasizzo and Kopitar-Jerala, 2020).

### Post-Translational Modifications

Post-translational modifications include phosphorylation, ubiquitination, acetylation, nitrosylation, glycation, and many others. These modifications alter protein function and signaling. AD-related proteins like APP, Aβ, tau, beta-secretase 1 undergo post-translational modifications (Marcelli et al., 2018). Perhaps the most recognized post-translational modification in AD is the hyperphosphorylation of tau. Similarly, tau can undergo the post-translational modification known as O-linked-*N*-acetylglucosaminylation (O-GlcNAcylation) (Arnold et al., 1996), and reduced O-GlcNAcylation of tau is thought to allow for increased tau hyperphosphorylation (Smet-Nocca et al., 2011; Yuzwa et al., 2012). O-GlcNAcylation occurs on nucleoplasmic and cytoplasmic proteins and relies on glucose and the hexosamine biosynthetic pathway and is ubiquitously expressed in rodent and human brain (Taylor et al., 2014). The combination of reduced CBF slowing the rate of glucose delivery and decreased GLUT-1 receptors (Simpson et al., 1994; Liu et al., 2008) hamper the availability of glucose to be utilized in the brain (Mosconi, 2005). Some studies have reported a reduction in O-GlcNAcylation in AD brain (Liu et al., 2004, 2009; Zhu et al., 2014; Pinho et al., 2018), while others have reported the opposite (Griffith and Schmitz, 1995; Förster et al., 2014). Forebrain-specific loss of the enzyme, O-GlcNAc transferase, required for O-GlcNAcylation, in adult mice leads to progressive neurodegeneration, including widespread neuronal cell death, neuroinflammation, increased production of hyperphosphorylated tau and amyloidogenic Aβ-peptides, and memory deficits (Wang et al., 2016). Furthermore, human cortical brain tissue from Alzheimer’s disease patients has significantly reduced levels of O-GlcNAc transferase (Wang et al., 2016). Overexpressing neuronal O-GlcNAc transferase in aged mouse brain improved learning and memory (Wheatley et al., 2019). Interestingly, diminished O-GlcNAcylation strongly correlates with mitochondrial abnormalities and loss of cell viability in AD (Pinho et al., 2018). O-GlcNAcylation has been postulated to be the missing link between metabolism and immune responses in AD (de Jesus et al., 2018). Future studies should further characterize post-translational modifications of proteins at various stages of AD (Kelley et al., 2019) and incident dementia.

## The Human Element of Alzheimer’s Disease

**FIGURE 2:**
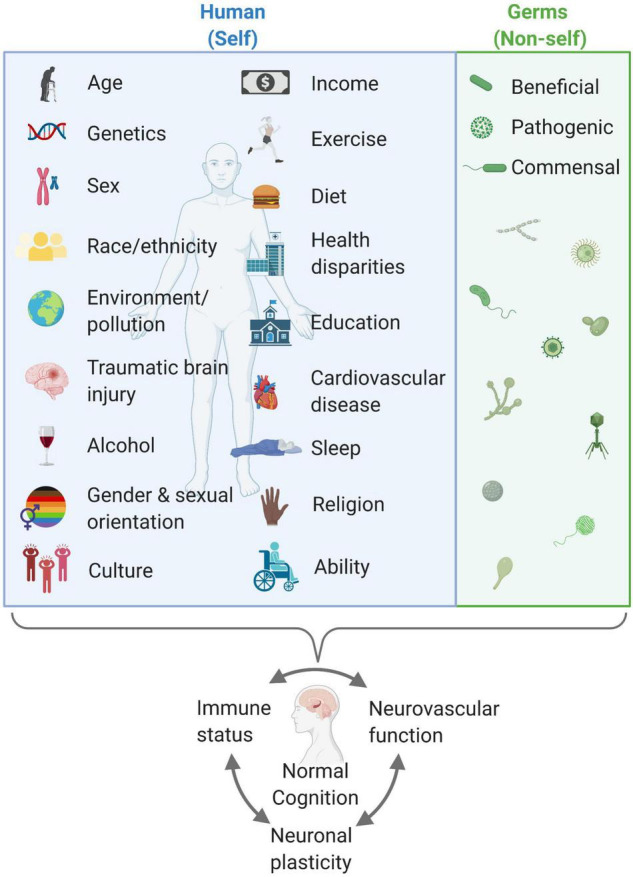
Humans are complex, variable organisms who sum up to have differential neurovascular function, immune status, and neuronal plasticity that impacts cognitive status (blue box). Furthermore, beneficial, pathogenic, and commensal germs also play a pivotal role. There are modifiable behaviors that may help in maintaining and promoting healthy cognition. A healthy balance of both self and non-self is key for a healthy body and mind. Created with BioRender.com.

Every person is different. There are many things that make us unique including age, genetic makeup, sex, race/ethnicity, environment/pollution, traumatic brain injuries, alcohol consumption, income, exercise, diet, health disparities, education, cardiovascular disease, or amount and quality of sleep, etc. In the case of AD, there are known genetic variants causing ADAD, and several identified genetic risk factors for sporadic AD (Longhe, 2020). Females are more likely than males to develop AD (Longhe, 2020). African, Native, and Hispanic Americans are more likely than non-Hispanic white Americans to develop AD (Longhe, 2020). Cardiovascular risk factors (e.g., hypertension, diabetes, atherosclerosis, and hyperhomocysteinemia), diet, exercise, and pollution influence AD risk (Nelson et al., 2016b; Longhe, 2020). Many AD cases may be attributed to modifiable risk factors (Baumgart et al., 2015; Andrews et al., 2020; Longhe, 2020; Wang et al., 2020). All of these factors impact neurovascular function and immune status and ultimately play into AD risk (Figure 2), as previously described (Baumgart et al., 2015; Nelson et al., 2016b; Longhe, 2020; Takeda et al., 2020). Assessing and altering modifiable risk factors for AD on an individualized basis may perhaps prevent or delay the onset and/or severity of AD. In section “Genetics,” we describe genes incriminated in AD, especially those implicated in neurovascular dysfunction and/or those impacting immunity.

### Genetics

#### Amyloid Precursor Protein

About 40 mutations in *APP* cause ADAD (Filley et al., 2007; Basun et al., 2008; Karch et al., 2014; Karch and Goate, 2015). ADAD accounts for ∼1% of all AD cases and exhibits early age of onset (<65 years of age) (Longhe, 2020). Several of these mutations lead to BBB breakdown and cerebrovascular pathology in humans and animal models (Grabowski et al., 2001; Ujiie et al., 2003; Kumar-Singh et al., 2005; Levy et al., 2006; Basun et al., 2008; Beckmann et al., 2011; Klohs et al., 2011, 2013; Merlini et al., 2011; Kruyer et al., 2015; Zarranz et al., 2016). The Icelandic mutation (e.g., A673T) of *APP* is less of a substrate for β-secretase resulting in less Aβ production. Even though carriers of this mutation in Finland had reduced Aβ plaques, there were reports of mild CAA suggesting that the vasculature may be less protected against Aβ accumulation than the brain parenchyma (Kero et al., 2013).

#### Presenilin

*PSEN1* and *PSEN2* encode presenilins 1 and 2, respectively, that act as aspartyl proteases to facilitate γ-secretase cleavage of APP to produce Aβ (Tanzi, 2012). There are 185 mutations reported in *PSEN1* and 13 mutations in *PSEN2* that have been identified in ADAD (Tanzi, 2012). These mutations increase the ratio of Aβ42:Aβ40 in the brain (Tanzi, 2012). *PSEN1* mutations lead to neurovascular pathology in humans including disruption of small cerebral blood vessels, degeneration of pericytes, BBB breakdown, and Aβ deposits in small cerebral arteries, arterioles, and capillaries (Armstrong, 2008; Niwa et al., 2013) and similar cerebrovascular dysfunction has been identified in animal models (Wen et al., 2005; Gama Sosa et al., 2010).

Case reports of *APP* (Suarez et al., 2019) and *PSEN* (Janssen et al., 2000; Stoychev et al., 2019) mutation carriers detailed the presence of severe infections, often times pneumonia, at the end stage of life. More comprehensive studies evaluating infection susceptibility and incident dementia rates in *APP* and *PSEN* mutation carriers are warranted.

#### Apolipoprotein E

There are three alleles of *APOE* in humans: *APOE* ε*2*, *APOE* ε*3*, and *APOE* ε*4*. *APOE* ε*4* is the strongest and most highly replicated risk factor for late-onset sporadic AD (Corder et al., 1993; Tanzi, 2012). A single *APOE* ε*4* allele increases an individual’s risk of AD by 3.7 and 12 times in carriers of two *APOE* ε*4* alleles, compared to *APOE* ε*3* carriers (Verghese et al., 2011). *APOE* ε*4* carriers have increased BBB damage, CAA, pericyte degeneration, and fibrinogen deposits in human brain (Zipser et al., 2007; Halliday et al., 2013, 2016; Hultman et al., 2013; Montagne et al., 2020). Recent studies in mice found that ApoE in brain pericytes regulate endothelial function by modulating basement membrane components (Yamazaki et al., 2020). *APOE* ε*4* increases the severity of infections such as herpes simplex virus type 1 (HSV-1) (Linard et al., 2020; Zhao et al., 2020), HIV-1 (Corder et al., 1998), and SARS-CoV-2 (Kurki et al., 2021) and worsens cognitive impairment.

#### Phosphatidylinositol Binding Clathrin Assembly Protein

Phosphatidylinositol binding clathrin assembly protein is a key player in Aβ clearance across the BBB (Zhao et al., 2015b). Approximately 20 single nucleotide polymorphisms (SNPs) in the non-coding region of PICALM have been associated with AD in several GWAS (Harold et al., 2009; Lambert et al., 2009; Carrasquillo et al., 2010; Corneveaux et al., 2010; Seshadri et al., 2010; Tanzi, 2012). Importantly, PICALM levels are reduced in AD brain endothelium, which correlated with elevated Aβ levels, Braak stage, and the level of cognitive decline (Zhao et al., 2015b).

#### Clusterin

Recent GWAS studies have identified a SNP within *CLU* on chromosome 8p21.1, rs11136000, that is significantly associated with sporadic AD (Harold et al., 2009; Lambert et al., 2009; Corneveaux et al., 2010; Seshadri et al., 2010; Tanzi, 2012). The C allele is an AD risk factor; whereas the minor T allele is protective and reduces the risk of AD by 16% (Harold et al., 2009; Lambert et al., 2009). Carriers of the CLU rs11136000 risk C allele exhibit reduced white matter integrity and hyperactivation of the prefrontal and limbic areas (Lancaster et al., 2014).

#### Triggering Receptor Expressed on Myeloid Cells 2

Triggering receptor expressed on myeloid cells 2 is expressed on microglia membranes and recognizes lipoproteins including APOE, phospholipids, and apoptotic cells and has been implicated in microglial phagocytosis and AD pathogenesis. Genetic variants in *TREM2* increase AD risk. In tauopathy, TREM2 deficiency exacerbates microglial responses to tau pathology (Bemiller et al., 2017). *TREM2*, together with *PLCG2* and *ABI3*, implicate innate immunity in AD pathophysiology (Sims et al., 2017). Increased *TREM2* expression in associated with the *CD33* risk allele (Chan et al., 2015).

#### Others

There are other genes including sortilin related receptor (*SORL1*), complement receptor 1 (*CR1*), and bridging integrator 1 (*BIN1*) that have been implicated in increasing AD risk (Lambert et al., 2009; Carrasquillo et al., 2010; Seshadri et al., 2010; Tanzi, 2012). *CR1* is a molecule of interest in the susceptibility and severity of infections, and autoimmune and inflammatory diseases (Khera and Das, 2009). Interestingly, individuals homozygous for minor *BIN1* SNP rs744373, an AD associated risk allele, had the highest mortality rate from SARS-CoV-2 compared to the major non-AD risk allele (Lehrer and Rheinstein, 2021a).

## Germs

### The Microbiome

There is growing evidence linking germs, whether beneficial, pathogenic, and/or commensal, to cognitive impairment and AD raising the infection hypothesis of AD (Panza et al., 2019; Seaks and Wilcock, 2020). Both alterations in gut microbiome (Tremlett et al., 2017; Vogt et al., 2017), and oral microbiome (Noble et al., 2009; Paganini-Hill et al., 2012; Kamer et al., 2015; Tremlett et al., 2017) have been connected to early cognitive dysfunction. Poor oral hygiene, oral inflammation, and tooth loss worsen with age and are risk factors for AD (Singhrao et al., 2014). Historically, it has been thought that the BBB protects the brain by preventing entry of “bugs” (Zlokovic, 2011; Zhao et al., 2015a). However, gut microbiota are known to influence BBB permeability in mice (Braniste et al., 2014), and several microbes disrupt BBB integrity in humans (Kang and McGavern, 2010).

Experimental studies have shown that gut microbiome regulate Aβ pathology (Minter et al., 2016, 2017; Dodiya et al., 2019), and that different pathogens elicit Aβ pathology by promoting its antimicrobial activity (Soscia et al., 2010; Kumar et al., 2016). There are differences in gut microbiome with *APOE* genotype in mice and humans (Tran et al., 2019). A recent study examined the gut microbiota on AD pathogenesis in an AD-like pathology with amyloid and neurofibrillary tangles (ADLPAPT) transgenic mouse model of AD, which showed amyloid plaques, neurofibrillary tangles, and reactive gliosis in the brain along with memory deficits (Kim et al., 2020). ADLPAPT mice had a loss of epithelial barrier integrity and chronic intestinal and systemic inflammation that differed from wild-type mice (Kim et al., 2020). Fecal transplant from wild-type to ADLPAPT mice ameliorated formation of Aβ plaques and tangles, gliosis and cognitive deficits, reversed abnormal macrophage-related gene expression in colon (Kim et al., 2020). Together these findings indicate that gut microbiome and systemic immune aberrations contribute to AD pathogenesis and suggest that correcting gut microbiome may be beneficial in AD (Kim et al., 2020).

### Incident Dementia, Infection, and Alzheimer’s Disease

Individuals who survive a critical illness such as respiratory failure or shock often have long-term cognitive impairment (Pandharipande et al., 2013). Bacterial pneumonia, congestive heart failure, dehydration, duodenal ulcer, and urinary tract infection are significantly higher among those with dementia (Phelan et al., 2012). More research is needed to fully understand the relationship between incident, sporadic, and genetic forms of dementia.

The concept that AD may stem from an infection was initially proposed by Dr. Fischer (Goedert, 2009; Buxbaum, 2017). Infectious agents including pneumonia, *Borrelia burgdorferi*, *Helicobacter pylori*, and HSV-1 have been reported in AD post-mortem brain tissue (Nicolson and Haier, 2009). *Porphyromonas gingivalis* have also been documented in AD brain tissue (Dominy et al., 2019). A cross-sectional study investigating associations between AD and prior infection with HSV-1, *Cytomegalovirus*, *Borrelia burgdorferi*, *Chlamydia pneumonia*, and *Helicobacter pylori* showed that patients with AD were significantly more likely than age-matched controls to have evidence of prior infection with *Cytomegalovirus* (odds ratio: 2.3) or *Chlamydia pneumonia* (odds ratio: 2.4) (Bu et al., 2015). AD subjects serum-positive for 4–5 microorganisms had the highest odds ratio (4.1) (Bu et al., 2015). Fungus was also observed in post-mortem brain tissue from AD subjects (Pisa et al., 2015a) and was found to localize around blood vessels in AD brain tissue (Pisa et al., 2015b). In AD, infectious agents likely enter the brain more easily due to an already leaky, disrupted BBB and cause even more detrimental effects.

#### Herpes

One of the earliest studies linking infection to AD used polymerase chain reaction and identified HSV-1 DNA, specifically the viral thymidine kinase gene, in control and AD brain samples (Jamieson et al., 1991). Interestingly, HSV-1 infections are found more often in *APOE* ε*4* carriers (Lin et al., 1996), which have increased BBB permeability (Montagne et al., 2020). Also, *APOE* ε*4* and HSV-1 together form an increased risk of AD than each one individually (Itzhaki et al., 1997). In post-mortem human brain HSV-1 DNA localizes within Aβ plaques (Wozniak et al., 2009b). Interestingly, HSV-1 induces tau phosphorylation via glycogen synthase kinase 3β and protein kinase A at several sites (e.g., serine 202, threonine 212, serine 214, serine 396, and serine 404) (Wozniak et al., 2009a).

Recent studies analyzed genes from almost 1,000 post-mortem brains and concluded that human herpes virus 6A (HHV-6A) and human HHV-7 were increased in AD (Readhead et al., 2018). However, HHV-6 detection across three independent AD brain repositories using RNA sequencing datasets and DNA samples extracted from AD and non-AD control brains were unable to identify an association between HHV-6 and AD (Allnutt et al., 2020). Aβ oligomers bind HSV-1 and human HHV- 6A and B surface glycoproteins, seeding β-amyloid deposition in 3D human neural cell cultures (Eimer et al., 2018). Furthermore, HSV-1 induced Aβ deposition in the subiculum of 5–6-week-old transgenic mice that overexpress Aβ (e.g., 5XFAD) that was not observed in wild-type littermate controls.

#### Pneumonia

Dementia patients have a 2-fold increased mortality rate from pneumonia (Foley et al., 2015), and pneumonia patients have elevated MMP-9 levels in their serum (Bircan et al., 2015), which is known to be linked to BBB breakdown (Halliday et al., 2013). Furthermore, there is long-term cognitive impairment following hospitalization for pneumonia (Girard et al., 2018). Pulmonary endothelial cells infected with *Pseudomonas aeruginosa* produced and released cytotoxic amyloid molecules with prion characteristics such as resistance to nucleases and proteases (Balczon et al., 2017). Lung endothelium infected with clinical isolates of either *P. aeruginosa*, *Klebsiella pneumoniae*, or *Staphylococcus aureus* produced and released cytotoxic amyloid and tau proteins (Balczon et al., 2019). *P. aeruginosa* infection elicited accumulation of detergent insoluble tau protein in mouse brain, inhibited synaptic plasticity, and impaired learning and memory (Balczon et al., 2019). Likewise, Aβ and tau isolated from *P. aeruginosa* infected patients and delivered intracerebroventricularly reduced dendritic spine density and reduce long-term potentiation (Scott et al., 2020). In another study, co-infection of lung with *Streptococcus pneumoniae* and influenza A virus leads to microglial activation in hypothalamus and expression of pro-inflammatory cytokines including tumor necrosis factor α, interleukin-1β, interleukin-6, and C-C motif chemokine ligand-2 (Wang et al., 2018a).

Early studies identified that *Chlamydia pneumoniae* is present, viable, and transcriptionally active in areas of neuropathology in AD brain (Balin et al., 1998). More recent studies also demonstrate a relationship between *C. pneumoniae* and AD (Gérard et al., 2006; Paradowski et al., 2007). *C. pneumoniae* infection promoted neurovascular inflammation (MacIntyre et al., 2003). Astrocytes infected with *C. pneumoniae* had altered expression and activity of secretases involved in the generation of β-amyloid (Al-Atrache et al., 2019).

Perhaps the timeliest pneumonia-associated incident dementia is that induced by SARS-CoV-2. It is too soon to know the long-term consequences of SARS-CoV-2 on AD prevalence, age of onset, and/or severity.

## Anti-Amyloid Antibodies

Several studies have investigated the presence of Aβ autoantibodies in biofluids and changes in their levels in AD with mixed results. Aβ autoantibodies were detectable in plasma but did not correlate with the plasma Aβ levels or dementia development in a cohort of 365 subjects (Hyman et al., 2001). An examination of 20 subjects found that Aβ autoantibody levels of the IgG1 and IgG3 subclass were lower in patients with posterior cortical atrophy and evidence of AD (Dorothée et al., 2012). Furthermore, the titer of anti-Aβ_42_ peptide autoantibodies were lower in AD than elderly control serum (Weksler et al., 2002). Another investigation measured Aβ autoantibody production at the cellular level and found that both healthy and AD patients have B cells that produced antibodies binding Aβ_40_ monomers and Aβ_42_ protofibrils and that number of Aβ_42_ antibody producing cells were higher in AD (Söllvander et al., 2015). Aβ autoantibodies in CSF were increased during CAA-related inflammation (Piazza et al., 2013; Crosta et al., 2015).

The link between Aβ autoantibodies and CAA are remarkable, as AD patients treated with anti-Aβ monoclonal antibody treatments often have a side effect of amyloid-related imaging abnormalities (ARIA) in the form of vasogenic edema and hemorrhage (Sperling et al., 2011; Ferrero et al., 2016; Mintun et al., 2021). In two large Phase 3 studies, 41% of patients receiving a higher dose of aducanumab developed iatrogenic ARIA, compared to 10% of controls (Salloway et al., 2022). ARIA could be especially problematic for patients with vascular dysfunction (e.g., cerebral small vessel disease, CAA, and *APOE* ε*4* carriers) (Sperling et al., 2011).

There have been a few studies measuring autoantibodies to other amyloids. Tau autoantibodies were detected in AD and non-demented control plasma but there was no significant difference between cohorts or correlation between tau autoantibodies and cognitive impairment (Yu et al., 2020). Interestingly, autoantibodies to tau, α-synuclein, microtubule associated protein-2, and others have been detected in the plasma of Gulf War veterans with myalgic encephalomyelitis/chronic fatigue syndrome (Abou-Donia et al., 2020). TDP-43 autoantibodies have been measured and found to be decreased (Nielsen et al., 2021) or increased (Conti et al., 2021) in ALS patient plasma or serum, respectively. More research is needed to understand the role and connections between amyloids, autoantibodies to amyloids and their impact on immune status.

## The Battle Between Humans and Germs

### Peripheral Amyloid Hypothesis to Cognitive Impairment and Alzheimer’s Disease

It has long been thought and taught that amyloids such as Aβ and tau are produced predominantly in the brain, and that impaired clearance of amyloids, rather than production drives accumulation in the brain (Ramanathan et al., 2015; Nelson et al., 2016a,^2017^). However, with the scientific growth of omics studies such as Human Protein Atlas (see footnote 1) (Uhlén et al., 2015), data is becoming more readily available suggesting the presence of amyloid genes and proteins in peripheral organs. For example, RNAseq studies from the Betsholtz lab in mice^2^ (He et al., 2018; Vanlandewijck et al., 2018) and from the Human Protein Atlas in humans show that MAPT gene is expressed in lung endothelial cells. We recently confirmed that tau is indeed expressed in lung capillary endothelial cells, is released in response to *P. aeruginosa* infection, and is able to seed neuronal tau (Choi et al., 2021).

In addition to the lung, podocytes in the kidney express tau (Xu et al., 2015). Parabiosis mouse studies found that the kidneys are involved in the clearance of tau from the circulation (Wang et al., 2018b). A large cohort study with 329,822 residents of Stockholm found that dementia occurred more frequently with poor kidney function assessed by higher estimated glomerular filtration rate (Xu et al., 2021). Transgenic mice that only express Aβ in the liver had capillary dysfunction, increased lipoprotein-Aβ entry into brain, neuroinflammation, and neurodegeneration (Lam et al., 2021).

**FIGURE 3:**
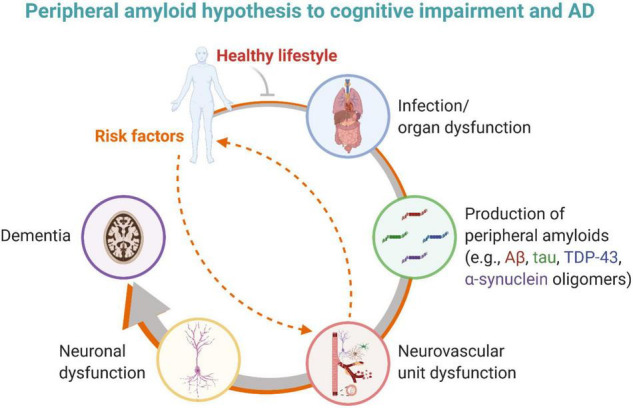
The peripheral amyloid hypothesis to cognitive impairment and AD states that amyloids (e.g., Aβ, tau, and perhaps TDP-43) are produced in the periphery as an innate immune response to infection or organ dysfunction, inducing neurovascular dysfunction, neurodegeneration, cognitive impairment, and ultimately AD. An individual may have risk factors that provoke and promote infection and/or organ dysfunction or that directly impact neurovascular unit function. A healthy lifestyle may help mitigate this pathological cascade. Created with BioRender.com.

Questions remain about how peripherally produced amyloids enter the brain, whether across a disrupted BBB, transported across the BBB (e.g., RAGE, LRP1, LRP2, etc.) or through the choroid plexus. It is critical to determine if the amyloids (e.g., Aβ, tau, TDP-43, and α-synuclein) associated with AD brain pathologies are initially produced in the periphery rather than central nervous system, and/or if peripheral amyloids induce or trigger brain plaques and tangles. In the case that peripheral amyloids are causative to cognitive impairment in dementia and AD, knowing what kickstarted the production and where (e.g., which organ and why), could be critical to determining the right treatment path for patients ideally early at the MCI stage. Taking together these concepts, the peripheral amyloid hypothesis to cognitive impairment and AD states that amyloids (e.g., Aβ, tau, TDP-43, and α-synuclein) are produced in the periphery as an innate immune response to infection or organ dysfunction, inducing neurovascular dysfunction, neurodegeneration, cognitive impairment, and ultimately AD (Figure 3). An individual’s risk factors (e.g., genetic, vascular, and environmental) superimposed with infection or organ dysfunction may exacerbate this cascade to dementia. Modifying an individual’s lifestyle (e.g., diet, exercise) for the better may dampen or slow the progression to dementia. Future studies are warranted to fully vet this new hypothesis.

A major question that remains in the AD field is why there is a specific pattern of spread of tau and TDP-43 in the brain, and why Aβ does not. Studies investigating microvascular architecture and/or density, pericyte type and/or coverage, BBB tight junction abundance and/or expression, and other potential neurovascular unit alterations from the point of view that the culprits are attacking from the bloodstream are timely and important.

### Antimicrobial Peptides

Neurodegenerative diseases, including AD, share several common pathologies including alterations to the levels of oligomeric amyloids in the blood and/or CSF (Klein et al., 2001; Tokuda et al., 2010; Benilova et al., 2012; Pietroboni et al., 2019; Majbour et al., 2020; Montalbano et al., 2020; Singh et al., 2020). Studies have shown that several oligomeric amyloids (Voth et al., 2020) including Aβ (Soscia et al., 2010; White et al., 2014; Bourgade et al., 2015; Kumar et al., 2016; Eimer et al., 2018), tau (Kobayashi et al., 2008), α-synuclein (Tulisiak et al., 2019; Linard et al., 2022), superoxide dismutase-1 (Pasupuleti et al., 2009) and perhaps others, such as TDP-43 (Bandea, 2013), are antimicrobial peptides generated as an innate immune response. These studies and others have encouraged the formation of the antimicrobial protection hypothesis of AD (Moir et al., 2018). In support of this idea, synthetic Aβ reduces the growth of common pathogens by up to 200-fold *in vitro* (Soscia et al., 2010). Aβ peptide strongly inhibit the infectivity of *influenza A* virus (White et al., 2014) and HSV-1 (Bourgade et al., 2015) in cell culture. Pharmacologically lowering Aβ levels with tarenflurbil in early AD patients caused increased infections (Green et al., 2009). Human Aβ protects and increases host survival, in transformed cell culture and transgenic *C. elegans* and mouse infection models (Kumar et al., 2016). Aβ being an antimicrobial peptide is further supported by Aβ oligomers that bound HSV-1 and HHV-6A and B surface glycoproteins, seeding Aβ deposition in transgenic mice and in 3D human neural cell cultures (Eimer et al., 2018). Recent studies found that SARS-CoV-2 Spike S1 protein receptor binding domain theoretically binds to Aβ, tau, TDP-43, prion and α-synuclein by examining protein-protein interactions (Idrees and Kumar, 2021). Antimicrobial peptides are potent immunomodulators (Moir et al., 2018). Are they prions? Questions remain about whether antimicrobial peptides such as Aβ and tau are also prions and transmissible (Asher et al., 2020), whether amyloids are helpful/cytoprotective, harmful/cytotoxic, neither or both, and if their role is dependent on folding and conformation, and/or on post-translational modifications.

### Vaccinations

Provocative data support the idea that AD is perhaps amyloid, infection, and immune dependent. Several vaccines (e.g., for influenza, tetanus, diphtheria and pertussis, herpes zoster, and others) have been demonstrated to reduce the incidence of AD (Verreault et al., 2001; Gofrit et al., 2019; Amran et al., 2020; Klinger et al., 2021; Lehrer and Rheinstein, 2021b; Scherrer et al., 2021). Further research on this topic is of the essence.

## Final Thoughts

The substantial investment in AD research over the last century has generated an energy of hope for a resolution to the mystery of this devastating disease. The field has moved beyond Aβ plaques and tau tangles in the brain as the only causes of AD to a more open mindset seeking to identify the root cause of the disease. At the same time, the large body of research publications related to AD all seem to be converging and connecting in ways that link back to the original descriptions of the disease, including a role of Aβ and tau in the disease pathogenesis. However, this role might be different than originally thought. How amyloids, including Aβ and tau, are innate immune mediators generated in the periphery in response to infection or organ dysfunction should be further explored. Furthermore, more information is needed about the role of amyloids as antimicrobial peptides. Is there a difference in sequence and/or structure between the amyloids that are antimicrobial peptides vs. those that are cytotoxic? Are peripheral amyloids generated in response to an infection or organ dysfunction involved in the seeding of brain amyloid or are they the same amyloids accumulating in the brain? Is the tau generated by lung endothelial cells in response to *P. aeruginosa* that seeded neuronal tau (Choi et al., 2021) a possible contributor to sporadic AD?

One critical aspect that remains to be determined is if peripheral pathways known to contribute to incident dementia, similarly and/or synergistically contribute to AD. Perhaps it is time to move beyond the brain to the periphery for causes of AD, and start early, at the MCI stage. Because every person and their lifestyle are unique, individualized medicine may be essential to determine the root cause of MCI with the hopes of preventing AD. This is especially the case since several peripheral organs can generate amyloids related to dementia. Could it be that one person had kidney dysfunction, another had pneumonia, etc., and that these incidents initiate a pathological cascade to dementia and ultimately AD? If so, the prevention and treatment to AD will likely require a combination of therapeutics on a case-by-case basis.

Thankfully, there have been substantial advances in neuroimaging and biomarkers of AD key players. We can identify early neurovascular dysfunction by detecting BBB breakdown (e.g., BBB_*ktrans*_ DCE-MRI, albumin quotient in biofluids), pericyte injury (e.g., sPDGFRβ in biofluids), neurodegeneration (e.g., neurofilament-L in biofluids), and gliosis (e.g., GFAP in biofluids). This could be coupled with not only cognitive assessment but also a complete medical history of potential peripheral contributors, keeping in mind that incidents influencing AD pathogenesis may occur years prior to MCI onset. Additionally, there are commercially available sensitive assays for detecting peripheral amyloids, including Aβ and tau, in plasma or serum. However, the antibodies used in these assays may not be able to differentiate where the amyloids were produced. To answer this question, it might perhaps be interesting to use Aβ and tau positron emission tomography radiotracer imaging in the periphery to determine if an organ other than the brain could be responsible for elevated production of amyloids.

Are we missing the root cause of AD by looking predominately at the brain? The probable answer is yes. If damage to blood vessels is the first hit to the brain in AD, perhaps the cause of neurovascular dysfunction is being transported to the brain via the circulation. There are experts in incident dementia who have likely been thinking about the links between incident dementia and AD for many years. Recent research is tying the AD-associated amyloids Aβ and tau with incident dementia. Bridging experts in incident dementia and AD may accelerate new discoveries to know the true root cause of AD, ways to prevent disease development and how to treat it. The peripheral amyloid hypothesis to cognitive impairment and AD warrants further research and will require a collaborative team from diverse trainings and backgrounds to answer.

## Author Contributions

The author confirms being the sole contributor of this work and has approved it for publication.

## Author Disclaimer

The views and opinions expressed in this manuscript represent those of the author and do not necessarily reflect those of the National Institutes of Health, or AlzOut.

## Conflict of Interest

The author declares that the research was conducted in the absence of any commercial or financial relationships that could be construed as a potential conflict of interest.

## Publisher’s Note

All claims expressed in this article are solely those of the authors and do not necessarily represent those of their affiliated organizations, or those of the publisher, the editors and the reviewers. Any product that may be evaluated in this article, or claim that may be made by its manufacturer, is not guaranteed or endorsed by the publisher.

## Footnotes

1
www.proteinatlas.org/

2
https://betsholtzlab.org/VascularSingleCells/database.html
